# Effectiveness of tailored individual follow-up vs. standard one-day group course on health-related quality of life in patients with post COVID-19 condition: a randomized controlled trial

**DOI:** 10.3389/fresc.2026.1927300

**Published:** 2026-09-22

**Authors:** Marte Wilson, Henriette Michalsen, Audny Anke, Synne G. Pedersen, Marina Espinasse, Nina Langeland, Rebecca J. Cox, Pål Aukrust, Tuva B. Dahl, Axel Sandvig, Maja Wilhelmsen

**Affiliations:** 1 Department of Rehabilitation, University Hospital of North Norway, Tromsø, Norway; 2 Institute of Health and Society, Research Centre for Habilitation and Rehabilitation Model and Services (CHARM), Faculty of Medicine, University of Oslo, Oslo, Norway; 3 Department of Clinical Medicine, UiT—The Arctic University of Norway, Tromsø, Norway; 4 Development and Research Department (UtForsk), University Hospital of North Norway, Tromsø, Norway; 5 Department of Clinical Science, University of Bergen, Bergen, Norway; 6 Department of Medicine, Haukeland University Hospital, Bergen, Norway; 7 Norwegian Institute for Public Health, Oslo, Norway; 8 Influenza Centre, Department of Microbiology, Haukeland University Hospital, Bergen, Norway; 9 Reseach Institute of Internal Medicine, Oslo University Hospital, Rikshospitalet, Oslo, Norway; 10 Faculty of Medicine, University of Oslo, Oslo, Norway; 11 Department of Research and Development, Division of Emergencies and Critical Care, Oslo University Hospital, Oslo, Norway; 12 Department of Neuromedicine and Movement Science, Norwegian University of Science and Technology, Trondheim, Norway; 13 Department of Neurology and Clinical Neurophysiology, St Olav’s Hospital, Trondheim, Norway

**Keywords:** cognitive behavioral therapy, cognitive training, fatigue, graded activity, long covid, mental fatigue, randomized controlled trial, rehabilitation

## Abstract

**Background:**

Post COVID-19 condition (PCC), is characterized by a wide range of symptoms, including fatigue and cognitive problems. Symptoms may lead to disability in everyday life and reduced health-related quality of life (HRQoL). Evidence for effective rehabilitation strategies for this patient group remains limited.

**Objective:**

To examine the effectiveness of tailored individual follow-up compared with a standard one-day group course, both based on cognitive behavioral approach, in patients with PCC 6 months after the intervention.

**Methods:**

A single-center, parallel-group, superiority randomized controlled trial (1:1) was conducted among outpatients aged 18–65 years with PCC. Patients were randomized to either a rehabilitation program with individual follow-up over 6–8 weeks or a standard one-day multidisciplinary self-management group course. Both groups were introduced to graded activity based on principles from cognitive behavioral therapy (CBT) and were offered the app “My COVID rehabilitation”. The primary outcome was the between-group difference in patient reported HRQoL (EQ-5D-5L-index score) at 6 months. Secondary self-reported outcomes included fatigue measured with the Chalder Fatigue Scale, EQ-VAS, and symptom registration. Neuropsychological function was assessed with objective neuropsychological tests.

**Results:**

60 participants were randomized, the mean age was 46 years (22–66 years), and 43 (72%) were female. Outcome assessments were completed for the primary outcome for 29 participants in each arm. No significant between-group difference was found in the primary outcome (score difference EQ-5D-5L-index =0.07; 95% CI [-0.18,.04]; *p* = .11). The adjusted linear model confirmed the absence of significant difference between groups. No persistent significant group differences were observed for any secondary outcomes. Both groups showed statistically and clinically significant sustained improvements in fatigue and EQ-VAS, as well as significant reductions in the number of symptoms. Within neuropsychological test, both groups demonstrated significant improvement in episodic memory (RAVLT), and the standard course group additionally showed significant improvement in attention (CPT).

**Conclusion:**

Tailored individual follow-up was not more effective than a standard one-day group course in improving HRQoL in PCC, both based on principles of CBT. However, significant improvements in both groups were observed in several secondary outcomes, including some objective neuropsychological tests. Larger studies are needed to confirm these results.

**Clinical Trial Registration**: https://clinicaltrials.gov/study/NCT06085911, identifier NCT06085911.

## Introduction

Post COVID-19 condition (PCC), also called long COVID, is associated with a wide range of persistent symptoms affecting multiple organ systems. Fatigue, dyspnoea, and symptoms of cognitive dysfunction are considered the three most frequently reported symptoms (1) and because of persistent symptomatology, PCC is also associated with reduced work participation and impairments in social and daily functioning (2). As a result, people with PCC face significant reductions in health-related quality of life (HRQoL) (3). According to the WHO 6% develop PCC following SARS-CoV-2 infection (4), however, the global prevalence of developing PCC varies considerably (5).

The symptom profile of PCC shows considerable overlap with other post-infectious conditions (6), suggesting that rehabilitation approaches developed for these related conditions may be relevant. Evidence for effective treatment strategies, however, remains limited, and randomized controlled trials (RCT) are scarce (7, 8). Most rehabilitation studies have focused primarily on physical exercise interventions and physical functioning outcomes (7, 9, 10), whereas fewer RCTs have addressed self-reported questionnaires exploring HRQoL, fatigue, and cognitive symptoms (8, 11). Even fewer have explored cognitive training and neuropsychological function by structural assessments with performance-based neuropsychological tests, which are important to obtain objective functional results (12). A recent study demonstrated that a multimodal rehabilitation approach incorporating both physical exercise and cognitive training was effective in individuals with PCC (13). Both guidelines and clinical reviews recommend individualized follow-up as part of the management of PCC (7, 14).

PCC is a complex condition (15), and cognitive behavioral therapy (CBT) addresses this complexity by targeting both cognitive processes and behavioral activity patterns, making it a relevant component of rehabilitation (8, 16). A few interventions based on principles of CBT have been shown to be effective in improving fatigue and physical functioning in PCCs (17, 18), when the intervention modality is provided as tailored follow-up consultations. These studies, however, did not include objective neuropsychological assessments.

As no national guidelines for the rehabilitation of PCC patients existed in Norway, a group-based course for post-viral fatigue syndrome/myalgic encephalopati (ME) was offered, providing education on fatigue and self-management strategies such as pacing; a strategy to balance activity and rest to avoid overexertion and reduce symptom flare-ups (19). Previous research on such self-management programs providing a holistic treatment approach and education on pacing in groups found no additional effect on fatigue compared to treatment as usual (20). To promote self-management, the use of rehabilitation goals is useful (21). Educational material and goal management may be supplemented as a pamphlet with a workbook or as a mobile app. Apps have been shown to be useful in the treatment of PCC populations (22, 23).

Findings from previous studies indicate that PCC is a complex condition that may be treated by a multimodal intervention based on principles from CBT. However, how to combine treatment contents, and which delivery modality is most effective, is not clear. To the best of our knowledge, no RCTs have yet evaluated an activity intervention provided as tailored individual follow-up compared to a one-day group course assessing HRQoL, fatigue, and cognitive symptoms. The present RCT that also includes neuropsychological assessments, seeks to address these knowledge gaps. We hypothesized that the tailored individual follow-up intervention would have better effect 6 months after intervention than standard one-day group course with no individual follow-ups on self-reported HRQoL, fatigue, symptom burden and objective neuropsychological function.

## Methods

### Design

A single-center, two-armed, parallel-group, superiority RCT with a 1:1 allocation ratio was carried out at the outpatient regional PCC clinic at the University Hospital of North Norway (UNN). With a 15% expected dropout, a power of 80%, and a *p*-value of .05, the required sample size was estimated to be 31 participants in each group. For more details, see the protocol (24).

The trial was approved by the Regional Committee for Ethics in Medical Research (587293) and written informed consent was obtained from all participants. Data was stored securely, and only the research team had access to the data. Results were presented in line with the Consolidated Standards of Reporting Trials (CONSORT) reporting guidelines (25).

#### Recruitment

Patients with symptoms of PCC were referred to the regional PCC clinic by their general practitioner. Clinical assessment was provided by a physician, in addition to a physiotherapist or a psychologist. Individuals aged between 18 and 65 years were included if they met the inclusion criteria of (1) symptoms attributable to PCC affecting daily activities according to WHO's definition (4), (2) a positive COVID-19 test at the time of infection; either a rapid antigen test or PCR test, (3) neuropsychological symptoms including fatigue and cognitive difficulties and (4) ability and willingness to provide informed consent. Exclusion criteria were: (1) patients who did not want to attend planned physical study visits, (2) patients who were unable to complete surveys in Norwegian, and (3) patients with known chronic neurocognitive disease before COVID-19 or other diseases that could explain current symptoms.

Eligible patients received an SMS invitation. Oral consent was provided by phone, and written informed consent was collected at the first visit. Recruitment and treatment intervention took place from October 2023 to December 2024. The 6 months outcome assessments were finished in May 2025.

#### Randomization

Participants were block randomized with the computer program Research Electronic Data Capture (REDCap) to either the intervention group (individual follow-up) or the standard treatment group (group course). No blinding was performed except for a blinded statistical evaluation of effectiveness.

### Study interventions

The aim of both the intervention group (individual follow-up) and the standard treatment (group course) was to improve self-management for PCC (24). The patients were encouraged to graded activity, meaning that participants were advised to stabilize their activities before gradually increasing them over time, based on their own tolerance (pacing). All therapists involved in delivering the interventions were trained in principles of CBT. Participants in both groups were not restricted from receiving additional health care services outside the study setting.

#### Study intervention: tailored individual follow-up

The participants in the intervention group received four consultations of 1–1.5 h by a physiotherapist over 6–8 weeks with two-week intervals. One physical consultation was followed by three video consultations, in total 4.5 h of individual therapist-patient interaction. An interdisciplinary team was available for supervision and discussion. Symptoms, function in everyday life, sustaining factors, and resources were thoroughly assessed in the first consultation. Education on sustaining factors of PCC, based on principles of CBT (26), was then provided. A tailored rehabilitation plan was developed in collaboration with the patient to support recovery by graded activity and home-based activities. The home-based activities had three focus areas: daily routine, physical activity, and cognitive training. In dialogue with the physiotherapist, each participant set weekly personal goals within these focus areas to guide the participant's progress. During individual follow-up consultations, participants were supported in reflecting on their progress, adjusting goals as needed, and maintaining engagement with the intervention.

#### Standard treatment; one-day group course

The control group underwent standard treatment, a one-day multidisciplinary group course lasting 6 h (including breaks) at the outpatient clinic in the rehabilitation department at UNN. Each group consisted of 8–10 participants. The course provided general education on known sustaining factors of PCC, based on principles of CBT, as well as graded activity. In addition, it addressed the importance of three focus areas: daily routine, physical activity, and cognitive activity. Information was provided by a physician, a psychologist, an occupational therapist, and/or a physiotherapist. Exchange of experience between participants was also facilitated. No individual support or follow-up consultations were provided.

#### mHealth supplement: COVID rehabilitation mobile app

A mobile app named *My COVID Rehabilitation* (Norwegian: Min COVID Rehabilitering) was offered as a supplement to all participants in both groups. Both groups were invited to voluntarily download the app. The app was developed in collaboration with user representatives, the multidisciplinary health care team at UNN, and system developers at Northern Norway Health ICT. The app was a digital informational resource providing some educational content on the prognosis and sustaining factors associated with PCC. It also enabled users to set individualized goals based on activities within three focus areas: daily routine, physical activity and cognitive training. Users subsequently received daily reminders related to their selected goal. No feedback or social interaction was possible in the app.

### Data collection

Web-based questionnaires were sent out by e-mail at baseline, 3- and 6 months, and data were captured in REDCap. If the participants had any questions regarding the questionnaires, they were offered help by the research coordinator. Neuropsychological tests were carried out at the hospital at baseline and at 6 months.

At baseline, the patient reported demographic data and relevant background information: height, weight, time since infection, number of vaccines, medication, comorbidities, highest level of education, occupational status, and sick leave since infection. App usage was registered at 3- and 6 months.

Adverse events were recorded, and all data were securely stored and de-identified prior to analysis.

#### Primary outcome

The between-group difference in HRQoL at 6 months was assessed using the EQ-5D-5L- index score (27, 28). EQ-5D-5L is a validated generic scale (28), also applied and shown to be responsive to PCC (22, 29, 30). The five dimensions include mobility, self-care, usual activity, pain/discomfort, and anxiety/depression. Each dimension is assessed with one question, which has five levels of severity (“no”, “slight”, “moderate”, “severe”, and “extreme” problems) (27, 28). The Norwegian index score has a range from −0.453 to 1, where 0 equals death, and 1 is perfect health (31). A score difference of 0.07 is considered to be a clinically significant difference (24).

#### Secondary outcomes

*Health-related quality of life:* HRQoL measured by EQ-5D-5L was collected at all three time points. In addition to the index score, the EQ includes a Visual Analog Scale (EQ-VAS), where respondents rated their overall health status on a scale from 0 (the worst imaginable health) to 100 (the best imaginable health). This provides a global assessment of health status (32). The MCID for EQ-VAS is estimated to be 6.9 (pulmonary disease) (33).

*Fatigue:* Subjective cognitive symptoms and fatigue were explored using the Chalder Fatigue Scale (CFS) (18, 21) previously used in research on PCC. The scale is validated and reliable as a generic self-administered questionnaire for measuring the extent and severity of physical and mental fatigue (34, 35). Each of the 11 items was answered on a 4-point scale ranging from asymptomatic to maximum symptomology (36), which are summed to a total score ranging from 0 to 33. The fatigue scale has previously been used in research on PCC (18, 21). A reference Minimal Clinically Important Difference (MCID) value for CFS is estimated to be 2.3 points (37). The mental fatigue subscale evaluates cognitive functioning and consists of items 8–11 of the Chalder Fatigue Scale, with scores ranging from 0 to 12 (36).

*Symptoms:* Burden of symptoms was explored through the presence of thirteen common symptoms of PCC (binary): fever, tingling, headache, dizziness, palpitations, nausea, cough, vomiting, diarrhea, altered taste/smell, sleep disturbances, chest-pain, and muscle/joint pain. Patients also rated the severity of dyspnea and feeling down/depressed the last month compared to the last time they felt well on a 4-point scale, dichotomized to a 2-point scale: less than/as usual and more than usual last month. The questions have previously been used in COVID research and have been shown to detect change (24, 38).

*Neuropsychological Function:* A comprehensive neuropsychological assessment was conducted at baseline and repeated at 6 months. To measure objective cognitive function four clinically relevant tests were selected from the test battery to evaluate 3 domains for comparison between groups and within groups after 6 months:

1. Episodic memory: Rey Auditory Verbal Learning Test (RAVLT) (39–41)
2. Executive functions: Trail Making Test 4 (TMT 4) and the Inhibition trial of the Color-Word Interference Test (CWIT 3), both from the Delis-Kaplan Executive Function System (D-KEFS) (42, 43)
3. Attention: Conners Performance Test (CPT—III, omissions) (44)

Higher RAVLT scores, lower CWIT and TMT scores indicate better performance. For CPT, scores close to the mean (T-score 50) indicate better performance. Assessment sessions lasted approximately 90 min, and the tests were performed by a clinical psychologist or a trained health care professional under supervision.

### Statistical analyses

Data were analyzed with IBM SPSS version 29 and with SAS Enterprise Guide version 8.3 (SAS Institute Inc. 2020. Cary, NC). Descriptive statistics were used to describe the study population (mean, range, SD and percent). Between-group differences were examined using chi-square tests, independent-samples t-tests, or the non-parametric Mann–Whitney U test. Within-group differences were analyzed using paired t-tests or the non-parametric Wilcoxon signed-rank test.

For repeated categorical data the Cochran's *Q* test was applied. Details of the statistical analysis plan and statistical analyses can be found in Supplementary Materials.

The primary outcome EQ-5D-5L-index score at 6 months, was calculated using the Norwegian syntax of EQ-5D-5L-index (31) and analyzed by a statistician blinded to the intervention groups. The between-group difference was first tested with an independent samples t-test. Then, a linear regression model adjusted for baseline EQ-5D-5L-index score, gender, and age was applied. A one-sided *p*-value was aimed to be estimated for the effect of intervention, since the study is a superiority trial. The linear regression was checked using the common diagnostics methods (the distribution of residuals, influential observations) and was validated using the bootstrap method.

To analyze the effect on the intervention of temporal change on the mean CFS, we applied Generalized Estimating Equations (GEE) model adjusted for patients' age and gender and including time effect and time by intervention interaction (an intervention-specific time trend). The CFS data had 14 missing data points (about 8% of the total dataset). However, GEE method might not be robust when the dataset has missing points, when the missing mechanism is not completely random (MCAR) (45). We did not assume missing completely at random (MCAR), because this is a relatively strong assumption (46), and have therefore applied a multiple imputation using MI procedure in SAS with a fully conditional specification as the imputation method (47). For all other outcomes, missing data was excluded from the analysis and no models or imputation were performed.

The main objective of the neuropsychological analysis was to detect between-group differences among patients with PCC rather than to compare test scores with a healthy control group (48). Raw scores were therefore chosen for detecting change between groups and within groups from baseline to 6 months follow up (49). Given the limited sample size, the analyses were considered exploratory.

## Results

Data were collected from November 21, 2023, until May 10, 2025. A total of 162 patients with PCC were assessed for eligibility, 89 were invited to the study and of these, 60 individuals were included (Flow chart, Figure 1). 31 participants were allocated to the intervention group (individual follow-up) and 29 participants were allocated to standard treatment (group course). All participants completed the treatment intervention, except 1 participant, who withdrew from the intervention group. All questionnaires were filled out at baseline and one participant did not reply at 3- and 6 month assessments. A flow diagram for the primary outcome is presented in line with CONSORT guidelines, Figure 1.

**Figure 1:**
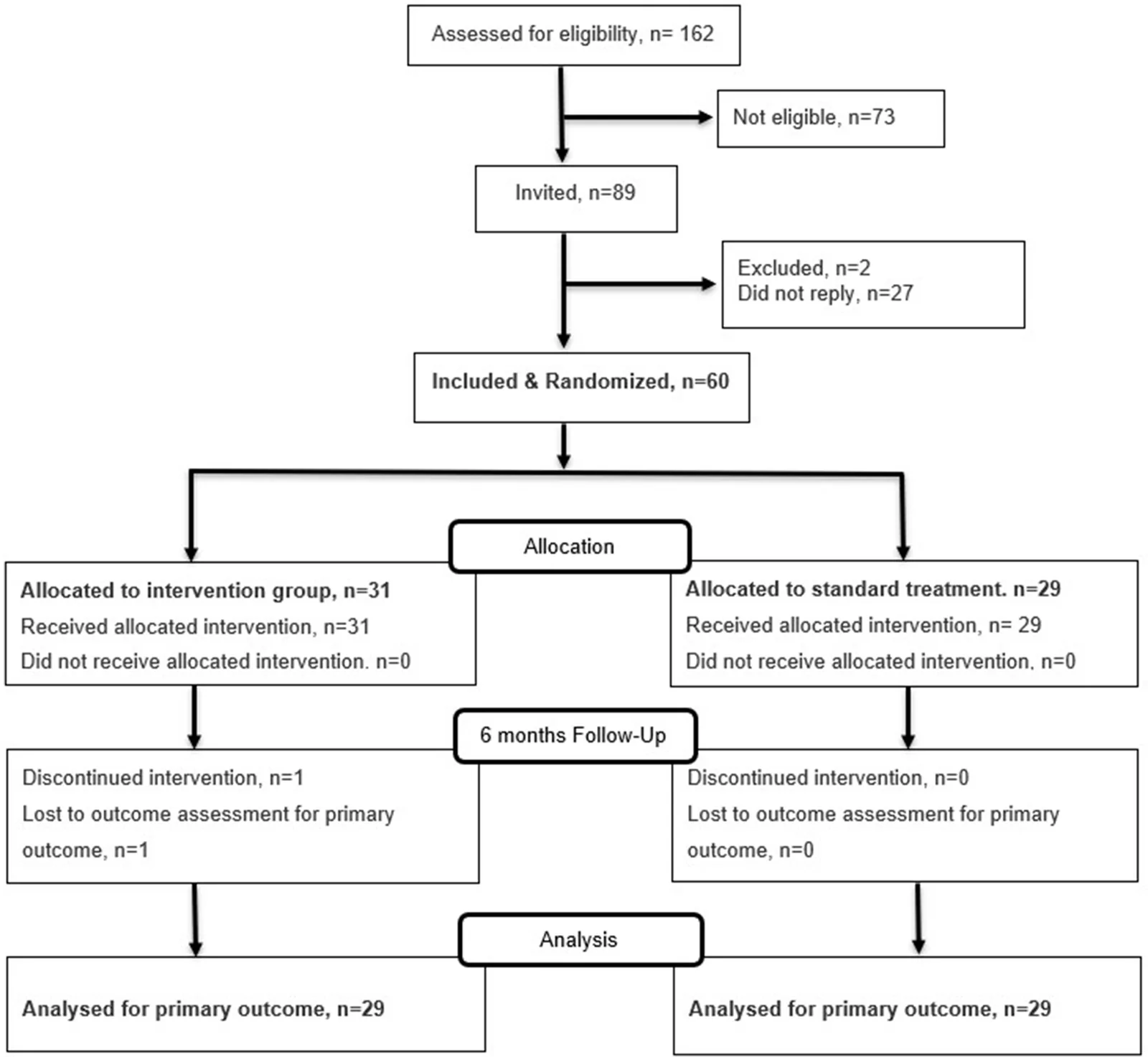
Flow diagram of study design, eligibility and inclusion for the primary outcome.

All participants (*n* = 60) completed neuropsychological tests at baseline, and 50 participants (83%) at 6 months. Of the 10 participants lost to follow-up, 8 were in the intervention group and 2 were in the standard course group. Nine participants cancelled neuropsychological tests due to reduced capacity, work or other plans. One participant in the intervention group was excluded from analyses because the test results were invalid.

Because of the low drop-out rate for primary outcome (*N* = 58), only per-protocol analyses were performed. No harms or unintended events were reported.

### Baseline characteristics

As shown in Table 1, the study included a sample of 60 individuals with PCC, of whom 43 were female (72%) and 17 were male (28%). The mean time since the acute SARS-CoV-2 infection was 1.9 years, range 0.4–4.6 years. The participants age ranged from 22 to 63 years, with a mean of 46 years. No statistically significant differences were detected between the groups at baseline. However, there was a tendency toward older age, higher BMI, and more comorbidities in the intervention group compared to the standard course group (Table 1).

**Table 1:** Participant demographics and medical history.

Characteristics	Standard treatment group	Intervention group	*p*-value
Allocation	29	31	
Age, years (mean/ range)	44 (22–62)	49 (28–63)	.*06*
Gender, *n* (%)			.*65*
Female	20 (69)	23 (74)	
Male	9 (31)	8 (26)	
BMI (mean/ range)	26.9 (20.1–42.8)	29.7 (19.8–51.7)	.*11*
Time of infection			
Education level, *n* (%)			
Secondary school	1 (3)	2 (7)	.*98*
High school	7 (24)	7 (23)	
University, 1–3 years	9 (31)	11 (35)	
University, 4 years or more	12 (42)	10 (35)	
Occupational status, *n* (%)			
Working/ studying	24 (83)	27 (87)	.*61*
Sick leave	3 (10)	3 (10)	
Not employed	2 (7)	1 (3)	
Absence from work, *n* (%)			
None	2 (7)	2 (6)	*1*.*0*
Reported absence	27 (93)	29 (94)	
Comorbidities, *n* (%)			
None	21 (72)	16 (52)	.*10*
Reported comorbidities	8 (28)	15 (48)	
Time since infection, (mean years/range)	1.8 (0.4–3.2)	2.0 (0.6–4.6)	.*54*
SARS-CoV-2 vaccine, *n* (%)			
None	3 (10)	2 (6)	
Vaccinated	26 (90)	29 (94)	
*1 Dose*	1	1	.*67*
*2 Doses*	5	7	
*3 Doses*	19	17	
*4 Doses*	0	2	
*5 Doses*	1	2	
Regular medication, *n* (%)			
None	8 (32)	13 (38)	
Reported medication	21 (68)	18 (62)	.*65*

BMI, body mass index.

Comorbidities (considered not to explain current symptoms) included: cardiovascular disease and/or hypertension (*n* = 1), rheumatic disease (*n* = 4), asthma or Chronic Obstructive Pulmonary Disease (*n* = 7), diabetes (*n* = 2) and congenital conditions (*n* = 2), hypothyroidism (*n* = 2), chronic sinusitis (*n* = 2), Attention-Deficit/Hyperactivity Disorder (*n* = 1), anxiety (*n* = 1), complex regional pain syndrome (*n* = 1), coeliac disease (*n* = 1), Ehler-Danlos syndrome (*n* = 1), irritable bowel syndrome (*n* = 2), chronic migraine (*n* = 2) and goiter (*n* = 1). The use of regular medications in relation to these disorders is shown in Table 1.

Participants were screened at baseline for possible conditions that may influence cognitive difficulties. Some reported reading and writing difficulties (*n* = 1), hearing difficulties (*n* = 1), learning difficulties (*n* = 1), dyslexia (*n* = 3), Attention-Deficit/Hyperactivity Disorder (*n* = 1), dysphasia (*n* = 1) and prior concussion (*n* = 1); reported by 4 patients in the intervention group and 3 patients in the control group.

### Primary outcome

For HRQoL (EQ-5D-5L-index score), no significant group difference was detected at the primary endpoint at 6 months (score difference = 0.07; 95% CI [−0.18, .04]; *p* = .11, one-sided t-test) (Table 3 and Figure 2). Similarly, the adjusted regression analysis showed no significant effect of treatment group allocation (*p* = .48) In addition, we found no effect of the covariates age and sex.

**Figure 2:**
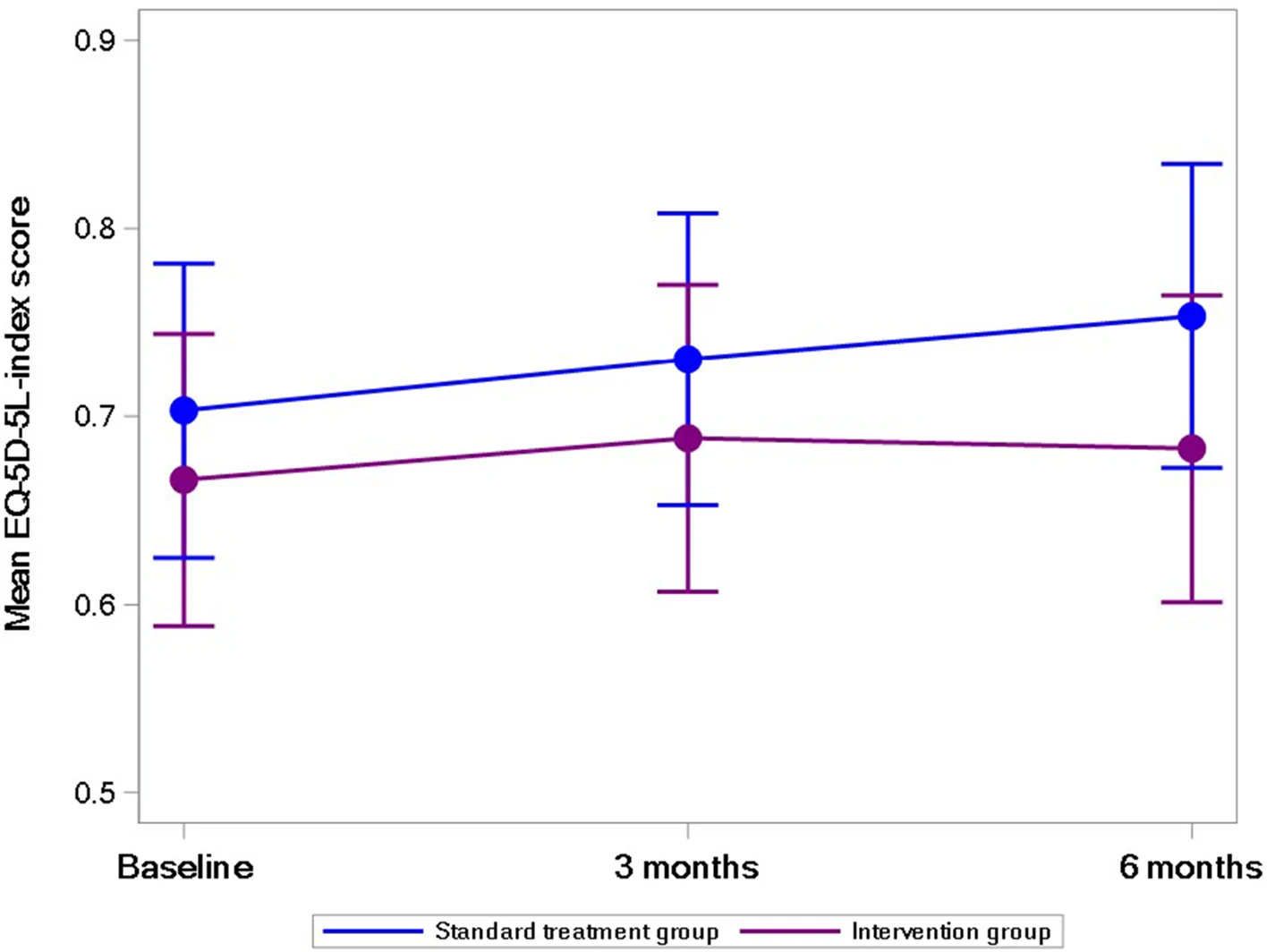
EQ-5D-5L-index score (range from −0.453–1) with 95% confidence interval (CI) at baseline, 3- and 6 months.

However, baseline EQ-5D-5L-index score was significantly associated with EQ-5D-5L-index score at 6 months (*p* < 0.001) (Table 2). The model is validated with bootstrap; the results can be found in the Supplementary Materials.

**Table 2:** Linear regression output for groups in EQ-5D-5L-index score at 6 months.

Variable	*β* (SE)	95% CI	*p*-value
Treatment group (intervention group compared to standard treatment)	−0.03 (0.05)	(−0.13–0.06)	.*48*
Covariates:			
Gender (female compared to male)	0.009 (0.05)	(−0.09–0.11)	.*11*
Age	−0.003 (0.002)	(−0.008–0.002)	.*18*
EQ-5D-5L- index score at baseline	0.67 (0.12)	(0.42–0.91)	*<*.*0001*

β, beta score; SE, standard error; CI, confidence interval.

### Secondary outcomes

An overview of secondary outcomes at baseline, 3- and 6 months with differences between groups are presented in Table 3.

**Table 3:** Overview of primary and secondary outcomes at baseline (0 months), 3- and 6 months included differences between groups.

Outcome measure	Time point (months)	*N*	Standard treatment group	Intervention group	*p*-value
Primary outcome					
EQ-5D-5L-index Mean (CI)	6	58	0.75 (0.67–0.83)	0.68 (0.6–0.76)	*.11* ^1^
Secondary outcomes					
EQ-5D-5L-index	0	60	0.70 (0.62–0.78)	0.67 (0.59–0.74)	.*36*
Mean (CI)	3	58	0.73 (0.65–0.81)	0.69 (0.61–0.77)	*.49*
	6	58	0.75 (0.67–0.83)	0.68 (0.6–0.76)	*.11* ^1^
EQ-VAS	0	58	46.3 (39.3–53.2)	46 (39.0–53.0)	.*92*
Mean (CI)	3	57	53.3 (44.8–61.7)	49.6 (41.4–57.7)	.*58*
	6	56	54.5 (44.6–64.3)^a^	56.6 (49.2–64.1)^a^	.*84*
Fatigue total: CFS	0	50	24.6 (23.0–26.2)	25.9 (24.4–27.4)	.*22*
Mean (CI)	3	58	21.2 (19.02–23.3)^a^	24.7 (23.1–26.3)^a^	.*01*
	6	58	21.0 (18.9–23.1)^a^	22.4 (20.0–24.8)^a^	.*19*
Fatigue mental: CFS, mean (CI)	0	50	8.1 (7.4–8.7)	8.8 (8.2–9.4)	.*08*
	3	58	7.4 (6.7–8.1)	8.9 (8.1–9.6)	.*02*
	6	58	6.9 (6.1–7.7)^a^	7.9 (7–8.9)^a^	.*06*
Number of Symptoms^2^	0	60	4.6 (3.6–5.5)	4.7 (4.0–5.5)	.*92*
Mean (CI)	3	58	4.0 (3.1–4.9)^a^	4.3 (3.4–5.2)	.*63*
	6	58	4.4 (3.5–5.3)	3.6 (2.5–4.6)^a^	.*22*
Symptom: depression^3^% (CI)	0	60	48.3 (30–66)	48.4 (30–66)	*1*.*0*
	3	58	39.3 (21–57)	33.3 (16–50)	.*64*
	6	58	34.5 (17–52)	44.8 (27–63)	.*42*
Symptom: breathlessnes^4^% (CI)	0	60	58.6 (40–77)	74.2 (59–90)	.*20*
	3	58	50 (34–69)	63.3 (46–80)	.*31*
	6	58	51.7 (34–70)	58.6 (40–77)	.*60*
Neuropsychological tests, mean (CI)					
Episodic memory: RAVLT total score	0	59	50 (47.1–53.7)	47.9 (43.9–51.9)	.*32*
	6	50	55.3 (52–58.5)^a^	50.7 (45.8–55.5)^a^	.*11*
Executive functions: TMT 4	0	57	75.2 (66.8–83.6)	70.6 (61.9–79.3)	.*31*
	6	49	70.5 (60.1–80.9)	74.5 (61–87.9)	.*90*
CWIT 3	0	58	58.0 (52.7–63.4)	55.1 (50.9–59.4)	.*74*
	6	49	53.27 (48.6–57.9)	54.6 (48.6–60.5)	.*78*
CPT-3 omissions	0	58	49.7 (47.2–52.2)	50.1 (47.1–53.2)	.*71*
	6	50	46.1 (45.1–47.2)^a^	49 (44.6–53.3)	.*83*

CI, 95% confidence interval; VAS, visual analog scale; CFS, Chalder Fatigue Scale; RAVLT, rey auditory verbal learning test; TMT, trail making test (subtest letter-number sequencing) and CWIT, color-word interference test (inhibition trial) from the Delis-Kaplan executive function system (D-KEFS); CPT—III, commissions from the conners continuous performance test, 3rd edition. Results presented as raw scores.

1 One sided *p*-value, t-tests.

2 Number of symptoms ranging from 0 to 13.

3 Symptom depression: feeling more depressed than usual (yes).

4 Symptom breathlessness: feeling more depressed than usual (yes).

a = statistically significant within-group differences from baseline.

#### Health-related quality of life

No significant group differences in EQ-5D-5L-index score were detected at 3 months, or EQ-VAS at 3- and 6 months. No significant within-group differences were detected in EQ-5D-5L-index score from baseline to 3- or 6 months. As shown in Figure 3, a significantly higher EQ-VAS score was observed at 6 months in both the standard course group and the intervention group compared to baseline. The mean EQ-VAS improvement exceeded the MCID for both groups, with 58% of participants in the intervention group and 45% in the standard course group reaching the MCID.

**Figure 3:**
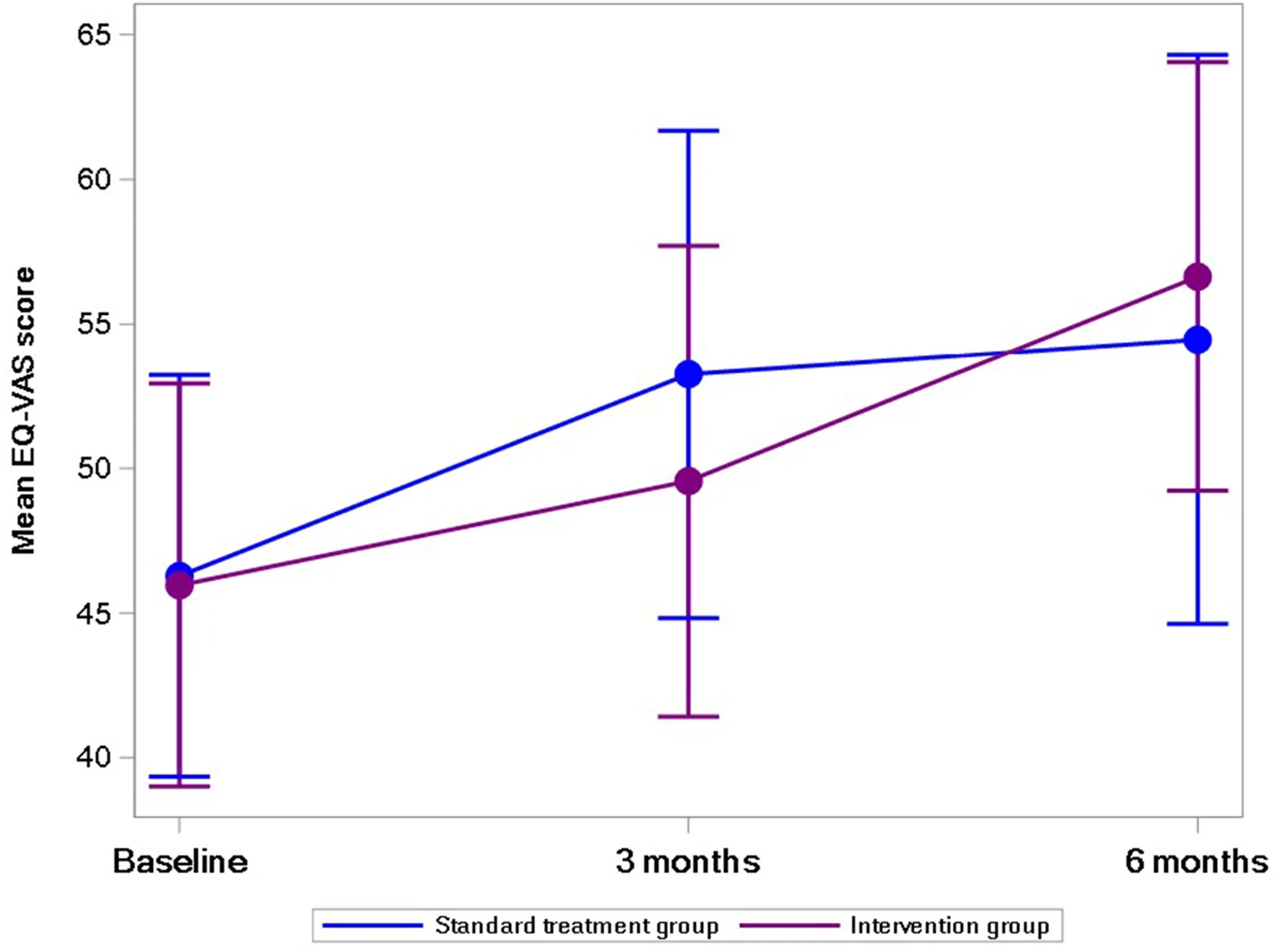
EQ-VAS score (range from 0 to 100) with 95% confidence interval at baseline, 3- and 6 months. CI, confidence interval; VAS, visual analog scale.

#### Fatigue

The GEE model showed that the Chalder fatigue score did not differ between the groups (time by treatment effect: *β* = −0.02; 95%CI [−0.12, 0.08]; *p* *=* .70), but both groups improved their fatigue score over time (time effect *β* = −0.15; 95%CI [−0.22, −0.08]; *p* < 0.001). Neither age nor sex had any effect on the fatigue score. The GEE model based on the imputed datasets agreed well with the same model run on the original data (see Supplementary Materials).

Within both groups there were statistically and clinically significant improvements in their total fatigue score, and statistically significant improvements in mental subscale score from baseline to 6 months. For total fatigue, statistically significant improvements were also observed from baseline to 3 months in both groups, whereas the standard course group exceeded the MCID at 3 months (Table 3 and Figure 4). At 6 months, 48% of the standard course group and 32% of the intervention group reached the MCID in total fatigue score.

**Figure 4:**
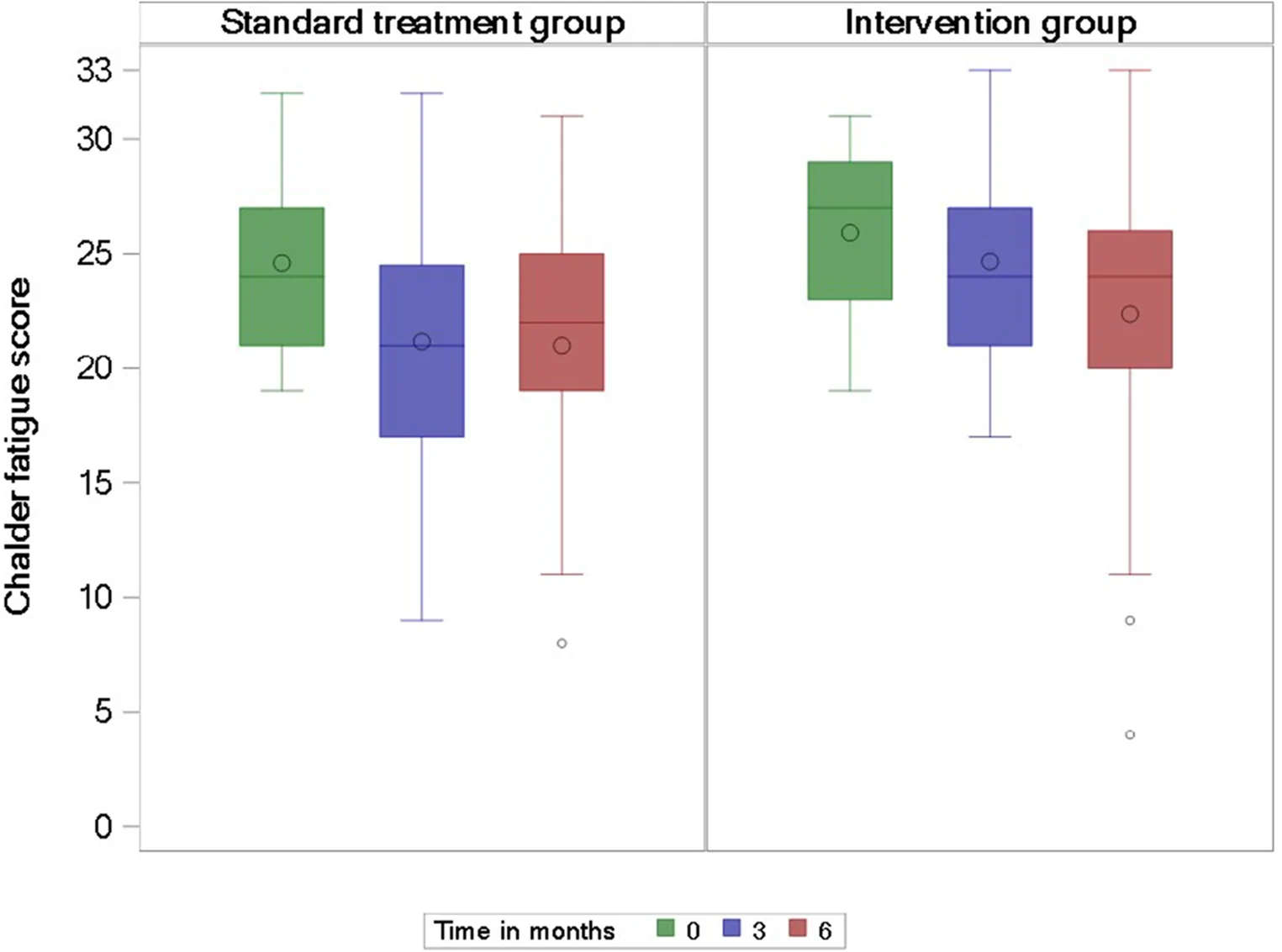
CFS total score at baseline, 3- and 6 months.

#### Symptoms

No significant between-group differences in the number of symptoms, depression and breathlessness were found. The number of symptoms decreased significantly from baseline to 3 months in the standard course group and from baseline to 6 months in the intervention group. No significant within-group differences in the proportion reporting depression or breathlessness were found across time.

#### Neuropsychological function

Objective neuropsychological test performance did not differ significantly between the groups at baseline or at 6 months (Table 3). A significant within-group improvement from baseline to 6 months was found in episodic memory (RAVLT total score) in both groups, and attention (CPT-3 omissions) in the standard course group. The average RAVLT scores in both groups were close to the population mean (40). Regarding executive functions, both groups scored slightly below the population mean on TMT 4 (50). For CWIT 3, both groups scored at the population mean (51). For CPT-3, both groups showed T-scores close to 50, corresponding to the population mean for the omission scores.

### Mobile application fidelity

No between group differences were found in terms of app use at 3 months (39% in standard course group; 63% in intervention group) and 6 months (28% in standard course group and 46% in intervention group).

## Discussion

In the present RCT of patients with PCC, we did not detect any differences between the intervention group (individual follow-up) and participants randomized to standard treatment (group course) for primary endpoint, HRQoL 6 months after baseline. For secondary outcomes, advanced analyses showed no significant group difference in fatigue, and no persistent group differences over time were observed for the other secondary outcome measures. Within both groups, however, significant, clinically important and persistent improvements were found in fatigue and overall health status, as well as significant reductions in the number of symptoms and episodic memory. In addition, attention improved in the standard course group. Both groups were introduced to graded activity based on principles of CBT. These findings may imply that an outpatient rehabilitation program with tailored individual follow-up was not more efficient than a standard one-day group course in patients with PCC. However, both groups significantly improved several important measures, including some objective neuropsychological measures.

Our findings differ from previous studies reporting superior outcomes following rehabilitation approaches with individualized follow-up based on principles of CBT in PCC with comparable baseline characteristics (17, 18). Nerli et al. (18) and Kuut et al. (17) compared individualized follow-up to care as usual provided outside the research institution. The follow-up interventions in these studies were conducted over a longer period and included more clinical follow-ups than in our study, which may explain the enhanced effect. In our study, standard group treatment was provided inside the research institution, but no individualized follow-up consultations were provided. The improvements observed in both groups and the lack of significant between-group differences may partly be explained by shared intervention components, including principles of CBT, physical activity and cognitive training. Both groups therefore received multimodal treatment, and a multimodal intervention has been shown to be effective in PCC (13). This is in contrast to the abovementioned studies by Nerli et al. (18) and Kuut et al. (17) which compared their intervention to usual care. In contrast to the present study, a self-management group course for patients with chronic fatigue was found to be less effective than care as usual (20), but this intervention was not explicitly based on CBT, physical activity and cognitive training.

Although not statistically significant, the baseline characteristics tended to be less favorable in the individualized group in the present study, including higher age, BMI and comorbidity burden, all of which are associated with worse PCC outcomes (52). Although the main analyses were adjusted for age, gender and baseline scores, these factors may still have influenced the results in the intervention group. PCC is a complex and heterogenic condition, and the study may therefore have been underpowered to detect clinically meaningful differences between two interventions containing therapeutic content, although patients in the standard course group received no guidance on how to implement treatment-related changes in their daily life over time. On the other hand, patients in the individualized program may have had different expectations of healthcare providers and their own role in the rehabilitation process compared with those attending the one-day group course. Participants in the one-day course may have assumed greater responsibility for their own rehabilitation and therefore successfully translated the gained knowledge into health-promoting behaviors in daily life. Spontaneous recovery may explain the improvements observed in both groups. However, the long symptom duration (mean 1.9 years) and the fact that more than 90% of participants reported work absence related to PCC suggest substantial functional impairment. We therefore argue that the likelihood of significant spontaneous recovery is reduced.

Different outcome measures have been applied in research on PCC. Nerli et al. (18) measured fatigue by using CFS total score, but their primary outcome measure was HRQoL measured with Short Form Health Survey 36 physical function subscale. In Kuut et al. (17), the primary outcome was the fatigue subscale of Checklist Individual Strength. Improvements showed both significant short- and long-term effects (17, 18). In this study, we were unable to detect significant between- or within-group differences of HRQoL using EQ-5D-5L-index scores. The EQ-5D-5L-index score was chosen as the primary outcome because it provides a brief, validated, and widely used measure that captures a broad range of functional domains (53). On the other hand, Carlile et al. (54) highlights limitations of the EQ-5D-5L in PCC, as its five dimensions may not fully capture important HRQoL impairments. This may explain the absence of significant changes in EQ-5D-5L-index scores.

Furthermore, factors not fully captured by the EQ-5D-5L may influence HRQoL outcomes in PCC. For example, sleep disturbances have been associated with poorer outcomes and lower quality of life in individuals with PCC. Exploring the mediating role of sleep disturbances was beyond the scope of this study but may partially explain our results (55, 56). In contrast to no between or within-group differences in EQ-5D-5L-index score in our study, both groups showed significant and clinically relevant improvements in self-reported overall health reported by EQ-VAS at both 3- and 6 months, aligning with previous findings suggesting that EQ-VAS may be more sensitive to symptom-related changes in PCC (29).

In the present study, high levels of subjective cognitive complaints, measured by self-reported mental fatigue scores, were observed in both groups. Significant improvements were observed in both groups at the 6 months follow-up. In contrast to high subjective cognitive complaints, results from objective neuropsychological tests were within the normal range. Because mental fatigue typically impairs processing speed and sustained attention (57), it was expected to influence performance on the neuropsychological measures used in this study. Within both groups, a statistically significant improvement was found in RAVLT scores (episodic memory), whereas CPT-3 omissions scores (attention) significantly improved within the standard course group. Findings from these two tests may support the improved subjective cognitive scores observed at the 6 months assessment, however no correlation analyses were performed to verify this relationship between subjective cognitive complaints and objective neuropsychological impairment. Previous studies report mixed findings examining the association between subjective cognitive complaints and objective neuropsychological impairment in PCC, with one reporting a significant association whereas the other found no significant association (58, 59).

Important aspects frequently affected in PCC include processing speed, sustained attention, working memory and cognitive fatigue (60, 61). The chosen battery includes clinically relevant tests for PCC, however the battery does not fully characterize PCC cognitive dysfunction. The discrepancy between improvements in both episodic memory and attention and the absence of improvement in executive function remains unclear and should be explored in future research. Interpretation of the neuropsychological test results should be made with caution, as the absence of a control group makes it difficult to distinguish rehabilitation-related cognitive improvement from practice effects, spontaneous recovery, or regression to the mean. Nine participants were lost to 6 months assessment and the lack of adjustment for age and sex in statistical analyses may have influenced the results.

A mobile app was developed as a supplement in the rehabilitation process in both treatment groups in the present study, based on feedback from our user representatives. There was no significant group difference in app use. There are inconsistent findings in the literature regarding the therapeutic effect of app-based rehabilitation for PCC (22, 23). However, the other studies used more advanced apps and included different content than our study. As the current study did not control for app use, we cannot rule out the possibility that the app itself contributed to therapeutic effects in both groups, potentially reducing the differences observed between the groups.

Symptom worsening after physical or mental exertion, also called post-exertional malaise (PEM) was not examined in our study. There is an ongoing debate about whether individuals with PEM should engage in graded activity interventions (62–65). Nevertheless, there is support for individually tailored programs, as they account for the complexity of the mechanisms underlying the symptoms. The current study recommended graded activity, where patients received information about pacing to avoid PEM. In the individual follow-up group, patients received tailored guidance, with the type of activity and dosage individually adjusted rather than standardized using fixed incremental steps. In contrast to previous reports, one-day group course, based on general recommendations and information including principles of CBT, showed similar results at both 3- and 6 months assessments compared to the individual follow-up intervention. General education, reassurance from an interdisciplinary team and interaction with other patients may have been just as therapeutic as a tailored individual plan with follow-ups, which may indicate that a one-day course might be sufficient in rehabilitation of PCC. Importantly, no adverse events related to the interventions were reported in either treatment group, suggesting that the treatments are safe for patients with PCC.

### Strengths and limitations

Strengths of this study include its design embedded in routine clinical practice, broad inclusion criteria, low dropout rates for the primary outcome, and the use of both patient-reported and objective outcome measures, including a comprehensive neuropsychological test battery.

Limitations include the single-center design, lack of blinding of participants and no registration of adherence to the home-based activities in the interventions. There is also an absence of an untreated control group and controls for concomitant care. Together this limits our ability to detect causal inference regarding intervention-specific effects vs. spontaneous recovery. Additionally, the multimodal nature of the interventions precludes identification of specific active components. The small sample size and power limit the generalizability of the results and the ability to detect true between-group differences. As the follow-up was limited to 6 months, the long-term effects of the treatment could not be evaluated in this study. Treatment fidelity, both from the therapist and patients, was not explored except for the proportion of app use.

### Practical implications

This RCT finds no superiority of individual follow-up compared to the standard treatment with a one-day group course in patients with PCC. Both groups showed improvements in fatigue, symptom count and EQ-VAS as well as some tests in neuropsychological assessments, reaching significant clinical differences at 6 months. These findings suggest that a one-day multidisciplinary group course based on principles of CBT may be a suitable treatment option for this patient population. Future research with larger sample sizes and inclusion of a control group without treatment is needed to determine the most effective interventions in PCC. Neuropsychological tests should be incorporated in larger future studies.

## Conclusions

In the present RCT, an intervention with tailored individual follow-up was not more effective than a standard one-day group course in improving HRQoL in PCC, both interventions were based on principles of CBT. Our study, however, showed a significant improvement, including fatigue, number of symptoms, overall health status and some neuropsychological measures, within both treatment groups. Larger studies are needed to confirm these results.

## Data Availability

The datasets presented in this article are not readily available to protect the anonymity of the participants. Requests to access the datasets should be directed to the corresponding author(s).
