# Supplementary material for: Effectiveness of tailored individual follow-up vs. standard one-day group course on health-related quality of life in patients with post COVID-19 condition: a randomized controlled trial
Source: Front Rehabil Sci. 2026 Sep 22;7:1927300. doi: 10.3389/fresc.2026.1927300 (PMC13638556; doi:10.3389/fresc.2026.1927300)
Supplement: Supplementary file 1 [file Datasheet1.pdf]

Contents

List of Figures 2

List of Tables 2

Supplementary information, tables and figures........ 4

Statistical analysis plan 4

Randomization........ 4

SAS codes for statistical models on HQOL and Chalder Fatigue Scale 5

GEE model on the Chalder Fatigue Scale 6

Missing data and multiple imputation of the Chalder Fatigue Scale data 7

References 8

Descriptive analysis of the secondary outcomes 10

List of Figures

Figure 1. A boxplot of Chalder mental score in each randomization arm, at 0, 3 and 6 months of follow-up. The boxplot for Standard treatment group at 3 months does not show the upper whisker, because multiple patients have reported the same score (8) at 3 months, so upper whisker (1.5IQR) and upper border of the box (75th percentile) coincided. 12

Figure 2. Boxplots of the VAS score in each randomization arm, at 0, 3 and 6 months of follow-up. 14

Figure 3. Boxplots of the total number of symptoms in each randomization arm, at 0, 3 and 6 months of follow-up. 15

Figure 4. The barplot of the percent of patients with depression in each randomization arm, at 0, 3 and 6 months of follow-up. 17

Figure 5. The barplot of the percent of patients with depression in each randomization arm, at 0, 3 and 6 months of follow-up. The percent of patients with depression per time points is broken down by gender. 17

Figure 6. The barplot of the percent of patients with breathing problems in each randomization arm, at 0, 3 and 6 months of follow-up. 19

Figure 7. The barplot of the percent of patients with breathing problems in each randomization arm, at 0, 3 and 6 months of follow-up. The percent of patients with depression per time points is broken down by gender. 19

List of Tables

Table 1.Parameter estimates of the covariate effects from the linear regression on the HQOL at 6 months follow-up, based on the 500 bootstraps from the original dataset. Bootstrap mean, standard error (SE), 2.5 and 97.5 percentiles for each estimate are presented 6

Table 2.Parameter estimates of the covariate effects from the GEE model on the Chalder Fatigue Scale, where missing data in Chalder Fatigue Scale are deleted. Standard error, 95% Confidence limits, critical value, and p-values for each parameter are presented 6

Table 3. Overview of the number of available and missing data points for Chalder Fatigue Scale data 7

Table 4. Missing data patterns in the Chalder Fatigue Scale for the Standard treatment group 7

Table 5. Missing data patterns in the Chalder Fatigue Scale for the Intervention group 7

Table 6. The output table of the MIANALYZE procedure in SAS, which combined the GEE models run on each imputation (50 imputation), using Rubin's rules. Supplementary information about multiple imputation analysis (e.g., variation within and between imputations, efficiency) is also presented. 9

Table 7. Overview table of the number of recorded and missing Chalder mental scores per time point. 11

Table 8. Mean, standard error and 95% Confidence interval for the Chalder mental score per randomization arm and time point (baseline, 3 months and 6 months of follow-up). 12

Table 9. Mean, standard error and 95% Confidence interval for VAS score per randomization arm and time point (baseline, 3 months and 6 months of follow-up). 13

Table 10. Overview table of the number of recorded and missing VAS scores per time point. 13

Table 11. Mean, standard error and 95% Confidence interval for the total number of symptoms per randomization arm and time point (baseline, 3 months and 6 months of follow-up). 14

Table 12. Overview table of the number of recorded and missing data of the total number of symptoms per time point. 15

Table 13. The count and percent per time point, of the patients with depression in the Standard treatment group. Confidence intervals for proportion of patients with depression are calculated as asymptotic Wald confidence intervals for a binomial value. 16

Table 14. The count and percent per time point, of the patients with depression in the Intervention group. Confidence intervals for proportion of patients with depression are calculated as asymptotic Wald confidence intervals for a binomial value. 16

Table 15. The count and percent per time point, of the patients with breathing problems in the Standard treatment group. Confidence intervals for proportion of patients with breathing problems are calculated as asymptotic Wald confidence intervals for a binomial value. 18

Table 16. The count and percent per time point, of the patients with breathing problems in the Intervention group. Confidence intervals for proportion of patients with breathing problems are calculated as asymptotic Wald confidence intervals for a binomial value. 18

Table 17. The count and percent at baseline and 6 months of follow-up of the patients that used the rehabilitation app in the Standard treatment group. Confidence intervals for proportion of patients that used the app is calculated as asymptotic Wald confidence intervals for a binomial value. 20

Table 18. The count and percent at baseline and 6 months of follow-up of the patients that used the rehabilitation app in the Intervention group. Confidence intervals for proportion of patients that used the app is calculated as asymptotic Wald confidence intervals for a binomial value. 20

The following sections of the supplemnetary materials present supplementary information , tables and figures related to the study. In these sections, the timepoints of the study can be referred to as T1, T2 and T3, corresponding to, respectively, baseline, 3 and 6 months

Supplementary information, tables and figures

Statistical analysis plan

The study plan included the following statistical analyses.

1) Comparison of the demographic characteristics (age, BMI, % of females, educational level, occupational status, absence from work, comorbidities, and time since COVID infection, number of vaccines and medication) between the two intervention groups. For the quantitative data such as age and BMI, we have planned to use t-test, while for the categorical data, such as the number of females in each group or earlier diabetes, we have planned to use a Chi-square test.
2) To investigate the effect of intervention on the HQOL, EQ-5D-5L-index score at 6 months, we have previewed to use a linear regression model with age, gender and baseline EQ-5D-5L-index score included in the model as potential confounders. The linear model was planned to be examined using conventional diagnostics methods and further validated using the bootstrap approach. No multiple imputation methods were previewed for the EQ-5D-5L-index score, since there were only 3 missing data points (at baseline and 6 months for the same patient, and at 6 months for another patient).
3) To investigate the effect of intervention on the Chalder Fatigue Scale over time (baseline, 3 months, and 6 months) we have thought to apply a model that is suitable for longitudinal type of data (a linear mixed model or GEE). In addition to time and intervention effects, this longitudinal model was planned to include age and gender. The selected longitudinal model was also planned to be rerun following the multiple imputation of the missing data in Chalder Fatigue Scale. The description of the multiple imputation analysis can be found below in the supplementary material.
4) The other secondary outcome measures (Chalder mental score, EQ VAS score, total number of symptoms, the presence of depression and breathing problems) and app use, were planned to be analyzed by primarily descriptive statistics and plots (mean/median, range, boxplots).

Randomization

Randomization table was prepared in the R programming environment (2021), using package *blockrand* (Snow, 2020), and then uploaded to REDCap web application, where actual allocation of patients took place. Participants were block randomized (block size 10) to the

intervention group (n= 32) or standard treatment group (n=30). No blinding was performed except for a blinded statistical evaluation of effectiveness.

SAS codes for statistical models on HQOL and Chalder Fatigue Scale

Here the SAS code use for the linear regression on the HQOL data at 6 months (coded as index_w26 variable). The baseline HQOL is coded as index_w0 variable.

```
proc glm data=covid.covid_qol plots(UNPACK)=diagnostics;
class randomisering(ref=”1”) gender;
 model index_w26=randomisering gender inclusion_age index_w0/ clparm;
 output out=cooks cookd=cooks_distance;RUN;
```

The bootstrap procedure was used to check the stability of the model results, if the same analysis would be repeated multiple times. The bootstrap was done 500 times by resampling the whole rows of the dataset.

```
%macro boostrap_linearmod;
 %do b=1 %to 500;
   %put NOTE: Starting resample b=&B;

     /* Bootstrap resample */
            proc surveyselect data=qol
                out=boot_data
                 method=urs
                 samprate=1 outhits
                          seed=%eval(1000+&b);
            run;

      data boot_data2;
        set boot_data;
     retain rowid 0;
        b = &b;
       run;

        proc glm data=boot_data2;
         class randomisering (ref="1") gender;
    model index_w26=randomisering gender inclusion_age index_w0/ clparm solution;
      ods output ParameterEstimates=params;
    RUN;
              data params;
                    set params;
                    b=&b;
              run;

        proc append base=all_qol_est data=params force;
              run;

      %end;
%mend;

%boostrap_linearmod

proc univariate data=all_qol_est;
class Parameter;
var Estimate;
output out=boot_univariate mean=Boot_mean  std=boot_se pctlpre=P_ pctlpts=2.5 97.5;
run;
```

Table 1.Parameter estimates of the covariate effects from the linear regression on the HQOL at 6 months follow-up, based on the 500 bootstraps from the original dataset. Bootstrap mean, standard error (SE), 2.5 and 97.5 percentiles for each estimate are presented

Parameter	Bootstrap mean	SE	2.5 percentile	97.5 percentile
Intercept	0.4277	0.1611	0.1144	0.7138
Intervention treatment group	-0.0334	0.0500	-0.1286	0.0671
Standard treatment group	0.0000	.	.	.
Female	-0.0110	0.0516	-0.1065	0.0945
Male	0.0000	.	.	.
Patient’s age at inclusion	-0.0032	0.0023	-0.0078	0.0007
EQ-5D-5L-index at baseline	0.6730	0.1569	0.3948	0.9967

GEE model on the Chalder Fatigue Scale

The table below shows the results of the GEE model on the Chalder Fatigue Scale, based on the original dataset.

Table 2.Parameter estimates of the covariate effects from the GEE model on the Chalder Fatigue Scale, where missing data in Chalder Fatigue Scale are deleted. Standard error, 95% Confidence limits, critical value, and p-values for each parameter are presented

Parameter	Estimate	Standard Error	95% Confidence Limits		Z	Pr > |Z|
Intercept	25.7371	2.4967	20.8437	30.6306	10.31	<.0001
Intervention treatment group	2.4727	1.0564	0.4020	4.5433	2.34	0.0193
Standard treatment group	0.0000	0.0000	0.0000	0.0000	.	.
Time (weeks)	-0.1315	0.0355	-0.2010	-0.0621	-3.71	0.0002
Time by treatment interaction	-0.0195	0.0508	-0.1191	0.0801	-0.38	0.7009
Female	0.8058	1.1740	-1.4953	3.1068	0.69	0.4925
Male	0.0000	0.0000	0.0000	0.0000	.	.
Patient’s age at inclusion	-0.0531	0.0523	-0.1556	0.0493	-1.02	0.3093

Missing data and multiple imputation of the Chalder Fatigue Scale data

The Chalder Fatigue Scale data had 14 missing data points (about 8% of the total), and their distribution over time is shown in the table below.

Table 3. Overview of the number of available and missing data points for Chalder Fatigue Scale data

Analysis Variable : Chalder Fatigue Scale			
**time**	**N Obs**	**N**	**N Miss**
Fatigue total T1	60	50	10
Fatigue total T2	60	58	2
Fatigue total T3	60	58	2

The patterns of missing data per randomization arm are shown in the tables below. In the standard treatment group, 25 patients had all 3 records of fatigues scores, 3 patients had only 2 last records, and 1 had only recorded fatigue at 6 months.

Table 4. Missing data patterns in the Chalder Fatigue Scale for the Standard treatment group

Missing Data Patterns Standard treatment group					
**Group**	**Fatigue total T1**	**Fatigue total T2**	**Fatigue total T3**	**Freq**	**Percent**
**1**	X	X	X	25	86.21
**2**	.	X	X	3	10.34
**3**	.	.	X	1	3.45

Table 5. Missing data patterns in the Chalder Fatigue Scale for the Intervention group

Missing Data Patterns Intervention group					
**Group**	**Fatigue total T1**	**Fatigue total T2**	**Fatigue total T3**	**Freq**	**Percent**
**1**	X	X	X	23	74.19
**2**	X	X	.	1	3.23
**3**	X	.	.	1	3.23
**4**	.	X	X	6	19.35

Below is the full code for multiple imputation of Chalder Fatigue Scale dataset, done in SAS Enterprise Guide. We applied the fully conditional specification (FCS) method, in which missing variables can be predicted from a set of other, non-missing variables, both continuous and categorical (Raghunathan et al., 2001; van Buuren, 2018).

The dataset used for the imputation is fatigue_wide (a wide format, where different time point's Fatigue Scale values are in separate columns). The 50 imputations are followed by 50 GEE models, and then by PROC MIANALYZE, where the results of multiple GEEs are combined using Rubin's rules.

References

Raghunathan TE, Lepkowski JM, Van Hoewyk J, Solenberger P. A multivariate technique for multiply imputing missing values using a sequence of regression models. Survey methodology. 2001 Jun;27(1):85-96.

van Buuren, S. (2018). Flexible Imputation of Missing Data, Second Edition (2nd ed.). Chapman and Hall/CRC. https://doi.org/10.1201/9780429492259


```
/*MULTIPLE IMPUTATION OF FATIGUE DATA*/

ods graphics on;
proc mi data=fatigue_wide seed=110925 out=fatigue_mi2 simple nimpute=50 round=0.1;
   class randomisering gender;

   fcs  reg(chalder_total_w0 = gender inclusion_age);
fcs    reg(chalder_total_w12 = randomisering  chalder_total_w0 gender
inclusion_age);
fcs    reg(chalder_total_w26 = randomisering chalder_total_w12 gender inclusion_age)
plots=trace;
var chalder_total: gender inclusion_age randomisering ;
run;

*Transpose the data into a long format;
data fatigue_migee2_prep;
set fatigue_mi2;
array fat (3) chalder_total:;
 do i = 1 to 3;
   fatigue_total=fat[i];
   time=i;

   output;
 end;
 run;

proc format;
 invalue weeks
1 = 0
2 = 12
3 = 26;
run;


data fatigue_migee2;
set fatigue_migee2_prep;
drop chalder_total: i ;
time_weeks = input(time, weeks.);
```

```
timeclass = put(time_weeks,15.);
time2 = time_weeks*time_weeks;
run;


ods trace off;
proc genmod data=fatigue_migee2 namelen=100;
by _imputation_;
class record_id gender randomisering (ref="1") timeclass;
model fatigue_total = randomisering  time_weeks randomisering*time_weeks
   gender inclusion_age/ dist=normaL LINK=identity maxiter=200 covb;
repeated subject=record_id/ withinsubject=timeclass
 type=exch;
 ods output GEEEmpPEst=gmparms parminfo=gmpinfo CovB=gmcovb ;
run;


proc mianalyze parms(classvar= level)=gmparms;
class gender randomisering;
modeleffects Intercept randomisering time_weeks randomisering*time_weeks gender
inclusion_age;
run;
```

The results of the GEE model, based on 50 imputed datasets are shown in the table below. For comparison, a table of GEE results based on the original dataset (with missing data), is inserted below.

Table 6. The output table of the MIANALYZE procedure in SAS, which combined the GEE models run on each imputation (50 imputation), using Rubin's rules. Supplementary information about multiple imputation analysis (e.g., variation within and between imputations, efficiency) is also presented.

Model Information	
**PARMS Data Set**	WORK.GMPARMS
**Number of Imputations**	50

Parameter	Variance			DF	Relative Increase in Variance	Fraction Missing Information	Relative Efficiency
	Between	Within	Total				
**Intercept**	0.177681	5.607925	5.789159	49997	0.032318	0.031345	0.999374
**Standard treatment group**	0	0	0	.	.	.	.
**Intervention treatment group**	0.121673	0.847926	0.972032	3005.9	0.146364	0.128257	0.997441
**Time (weeks)**	0.000081731	0.001397	0.001480	15454	0.059669	0.056432	0.998873
**Time by treatment interaction**	0.000303	0.003033	0.003341	5736.7	0.101832	0.092736	0.998149
**Female**	0.062010	1.049680	1.112930	15171	0.060256	0.056956	0.998862
**Male**	0	0	0	.	.	.	.
**Patient's age at inclusion**	0.000067148	0.002372	0.002440	62206	0.028877	0.028097	0.999438

Parameter	Estimate	Std Error	95% Confidence Limits		DF	Minimum	Maximum
**Intercept**	25.424542	2.406067	20.70862	30.14046	49997	24.521712	26.267898
**Standard treatment group**	0	0	.	.	.	0	0
**Intervention treatment group**	2.132132	0.985917	0.19899	4.06527	3005.9	1.394656	3.148808
**Time (weeks)**	-0.137566	0.038477	-0.21299	-0.06215	15454	-0.155749	-0.110338
**Time by treatment interaction**	-0.008439	0.057806	-0.12176	0.10488	5736.7	-0.050940	0.026769
**Female**	1.031757	1.054955	-1.03608	3.09960	15171	0.434371	1.637635
**Male**	0	0	.	.	.	0	0
**Patient's age at inclusion**	-0.048717	0.049400	-0.14554	0.04811	62206	-0.065654	-0.033918

Parameter	t for H0: Parameter=Theta0	Pr > |t|
**Intercept**	10.57	<.0001
**Standard treatment group**	.	.
**Intervention treatment group**	2.16	0.0307
**Time (weeks)**	-3.58	0.0004
**Time by treatment interaction**	-0.15	0.8839
**Female**	0.98	0.3281
**Male**	.	.
**Patient's age at inclusion**	-0.99	0.3241

Descriptive analysis of the secondary outcomes

Below are the descriptive tables and plots for the secondary outcomes: Chalder mental score (mental fatigue), VAS scores, total number of Symptoms, the presence of depression, and the usage of rehabilitation app.

Table 7. Overview table of the number of recorded and missing Chalder mental scores per time point.

Analysis Variable: Chalder mental score			
**time**	**N Obs**	**N**	**N Miss**
Chalder mental T1	60	50	10
Chalder mental T2	60	58	2
Chalder mental T3	60	58	2

Table 8. Mean, standard error and 95% Confidence interval for the Chalder mental score per randomization arm and time point (baseline, 3 months and 6 months of follow-up).

Analysis Variable: Chalder mental score						
**Treatment group**	**time**	**N Obs**	**Mean**	**Std Error**	**Lower 95% CL for Mean**	**Upper 95% CL for Mean**
Srandard treatment group	Chalder mental T1	29	8.0800	0.3158	7.4282	8.7318
	Chalder mental T2	29	7.4286	0.3311	6.7493	8.1078
	Chalder mental T3	29	6.9310	0.3842	6.1440	7.7180
Intervention group	Chalder mental T1	31	8.8400	0.2926	8.2362	9.4438
	Chalder mental T2	31	8.8667	0.3547	8.1412	9.5921
	Chalder mental T3	31	7.9310	0.4520	7.0052	8.8568

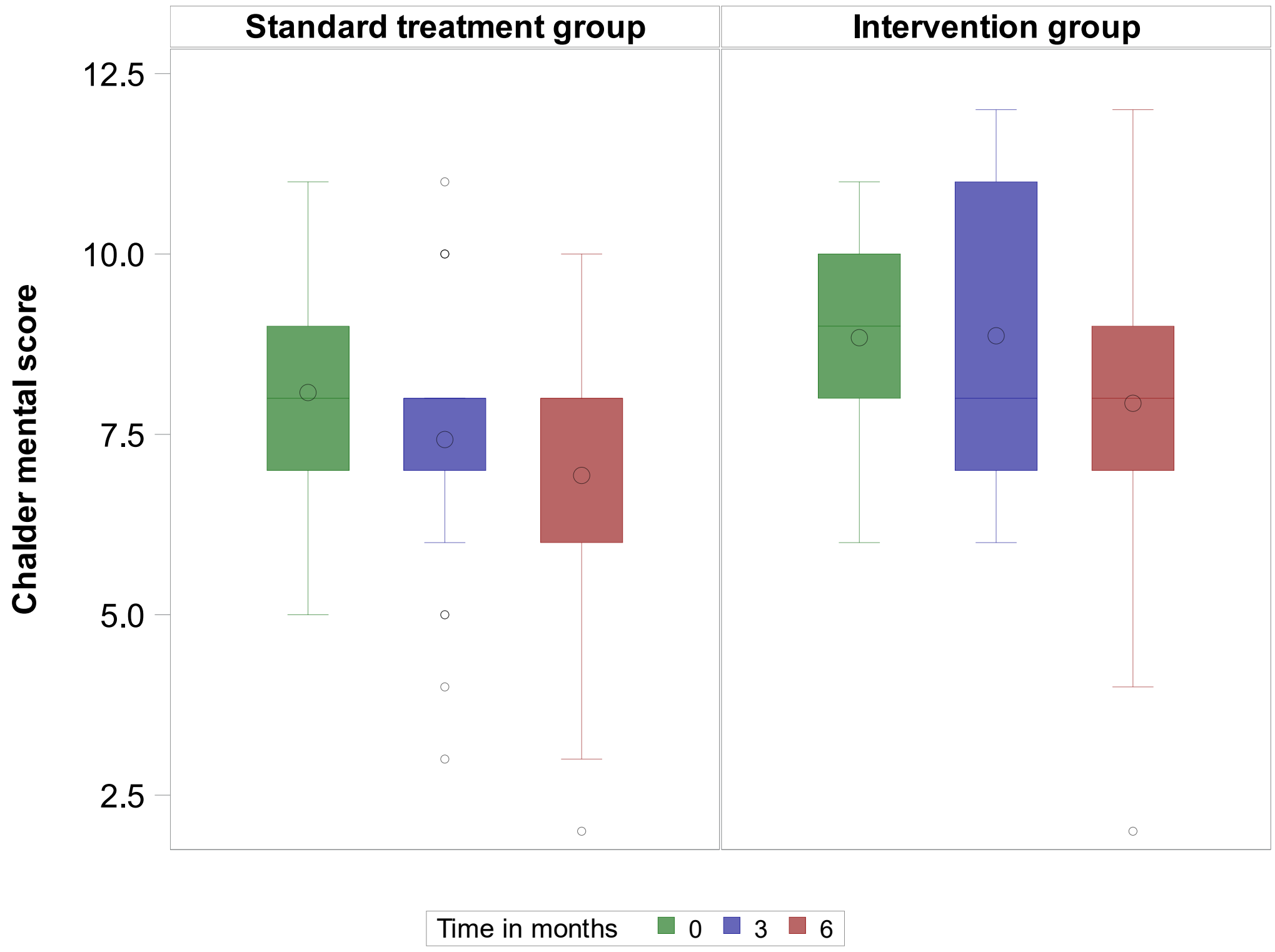

Figure 1. A boxplot of Chalder mental score in each randomization arm, at 0, 3 and 6 months of follow-up. The boxplot for Standard treatment group at 3 months does not show the upper whisker, because multiple patients have reported the same score (8) at 3 months, so upper whisker (1.5IQR) and upper border of the box (75th percentile) coincided.

Table 9. Mean, standard error and 95% Confidence interval for VAS score per randomization arm and time point (baseline, 3 months and 6 months of follow-up).

Analysis Variable: VAS						
**Group**	**NAME OF FORMER VARIABLE**	**N Obs**	**Mean**	**Std Error**	**Lower 95% CL for Mean**	**Upper 95% CL for Mean**
Srandard treatment group	EQ-VAS T1	29	46.2857	3.3892	39.3317	53.2397
	EQ-VAS T2	29	53.2593	4.1011	44.8292	61.6893
	EQ-VAS T3	29	54.4643	4.7932	44.6294	64.2991
Intervention group	EQ-VAS T1	31	45.9667	3.4081	38.9964	52.9369
	EQ-VAS T2	31	49.5667	3.9789	41.4290	57.7044
	EQ-VAS T3	31	56.6429	3.6111	49.2334	64.0523

Table 10. Overview table of the number of recorded and missing VAS scores per time point.

Analysis Variable: VAS			
**NAME OF FORMER VARIABLE**	**N Obs**	**N**	**N Miss**
EQ-VAS T1	60	58	2
EQ-VAS T2	60	57	3
EQ-VAS T3	60	56	4

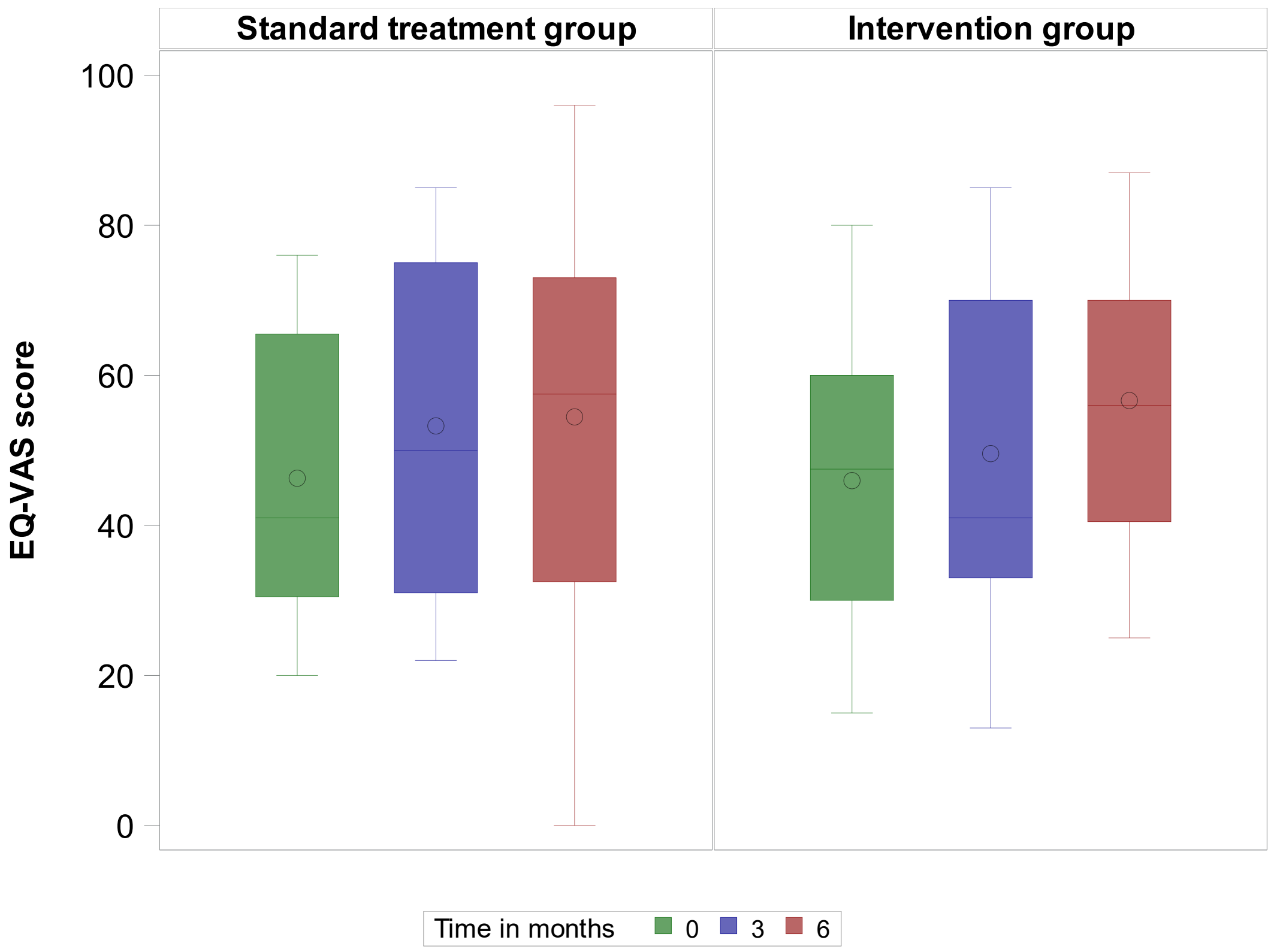

Figure 2. Boxplots of the VAS score in each randomization arm, at 0, 3 and 6 months of follow-up.

Table 11. Mean, standard error and 95% Confidence interval for the total number of symptoms per randomization arm and time point (baseline, 3 months and 6 months of follow-up).

Analysis Variable: total number of symptoms						
**Treatment group**	**NAME OF FORMER VARIABLE**	**N Obs**	**Mean**	**Std Error**	**Lower 95% CL for Mean**	**Upper 95% CL for Mean**
Srandard treatment group	Number of symptoms T1	29	4.5517	0.4564	3.6167	5.4867
	Number of symptoms T2	29	4.0000	0.4364	3.1045	4.8955
	Number of symptoms T3	29	4.3793	0.4450	3.4677	5.2909
Intervention group	Number of symptoms T1	31	4.7097	0.3686	3.9568	5.4626
	Number of symptoms T2	31	4.3000	0.4319	3.4166	5.1834
	Number of symptoms T3	31	3.5517	0.4903	2.5475	4.5560

Table 12. Overview table of the number of recorded and missing data of the total number of symptoms per time point.

Analysis Variable: total number of symptoms			
**NAME OF FORMER VARIABLE**	**N Obs**	**N**	**N Miss**
Number of symptoms T1	60	60	0
Number of symptoms T2	60	58	2
Number of symptoms T3	60	58	2

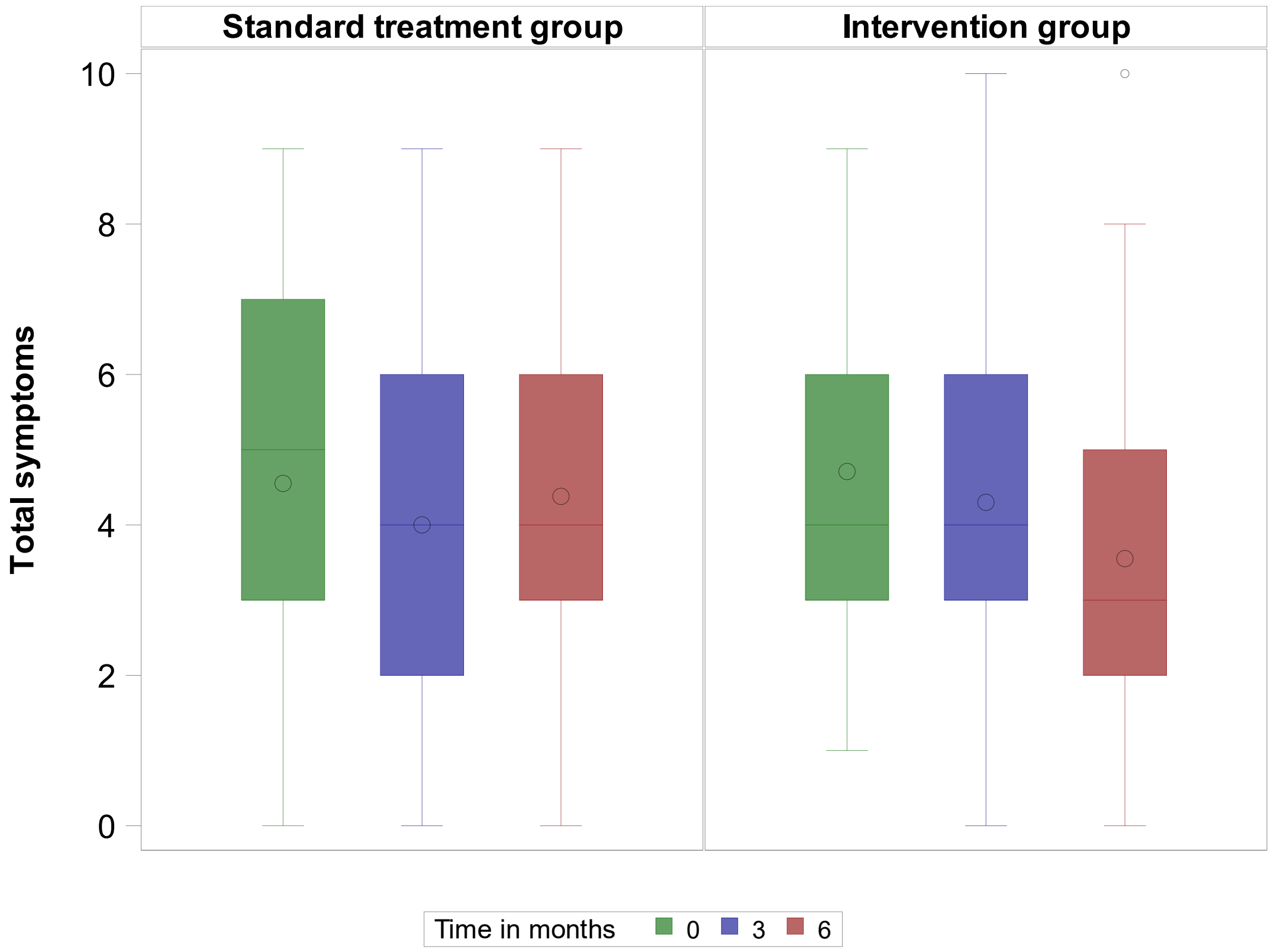

Figure 3. Boxplots of the total number of symptoms in each randomization arm, at 0, 3 and 6 months of follow-up.

Table 13. The count and percent per time point, of the patients with depression in the Standard treatment group. Confidence intervals for proportion of patients with depression are calculated as asymptotic Wald confidence intervals for a binomial value.

Time	Depression (no/yes)			Wald CI
**Frequency**<br>**Percent of the total per time point**	**0**	**1**	**Total**	
**Symptom: depression T1**	15<br>51.72	14<br>48.28	29	(0.3009, 0.6646)
**Symptom: depression T2**	17<br>60.71	11<br>39.29	28	(0.2120, 0.5738)
**Symptom: depression T3**	19<br>65.52	10<br>34.48	29	(0.1718, 0.5178)
**Total**	51	35	86	
**Frequency Missing = 1**				

Table 14. The count and percent per time point, of the patients with depression in the Intervention group. Confidence intervals for proportion of patients with depression are calculated as asymptotic Wald confidence intervals for a binomial value.

Time	Depression (no/yes)			Wald CI
**Frequency**<br>**Percent of the total per time point**	**0**	**1**	**Total**	
**Symptom: depression T1**	16<br>51.61	15<br>48.39	31	(0.3080, 0.6598)
**Symptom: depression T2**	20<br>66.67	10<br>33.33	30	(0.1646, 0.5020)
**Symptom: depression T3**	16<br>55.17	13<br>44.83	29	(0.2673, 0.6293)
**Total**	52	38	90	
**Frequency Missing = 3**				

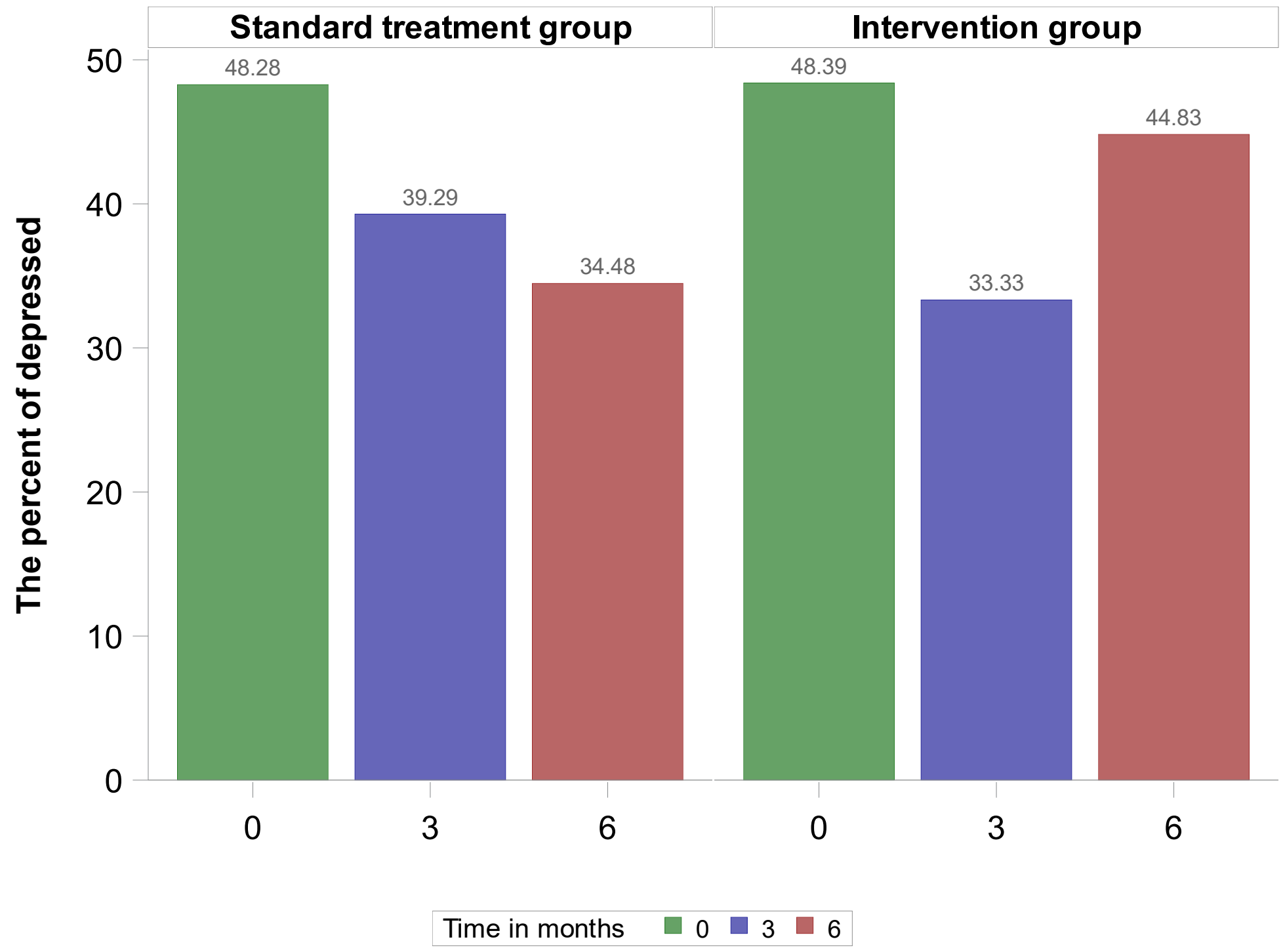

Figure 4. The barplot of the percent of patients with depression in each randomization arm, at 0, 3 and 6 months of follow-up.

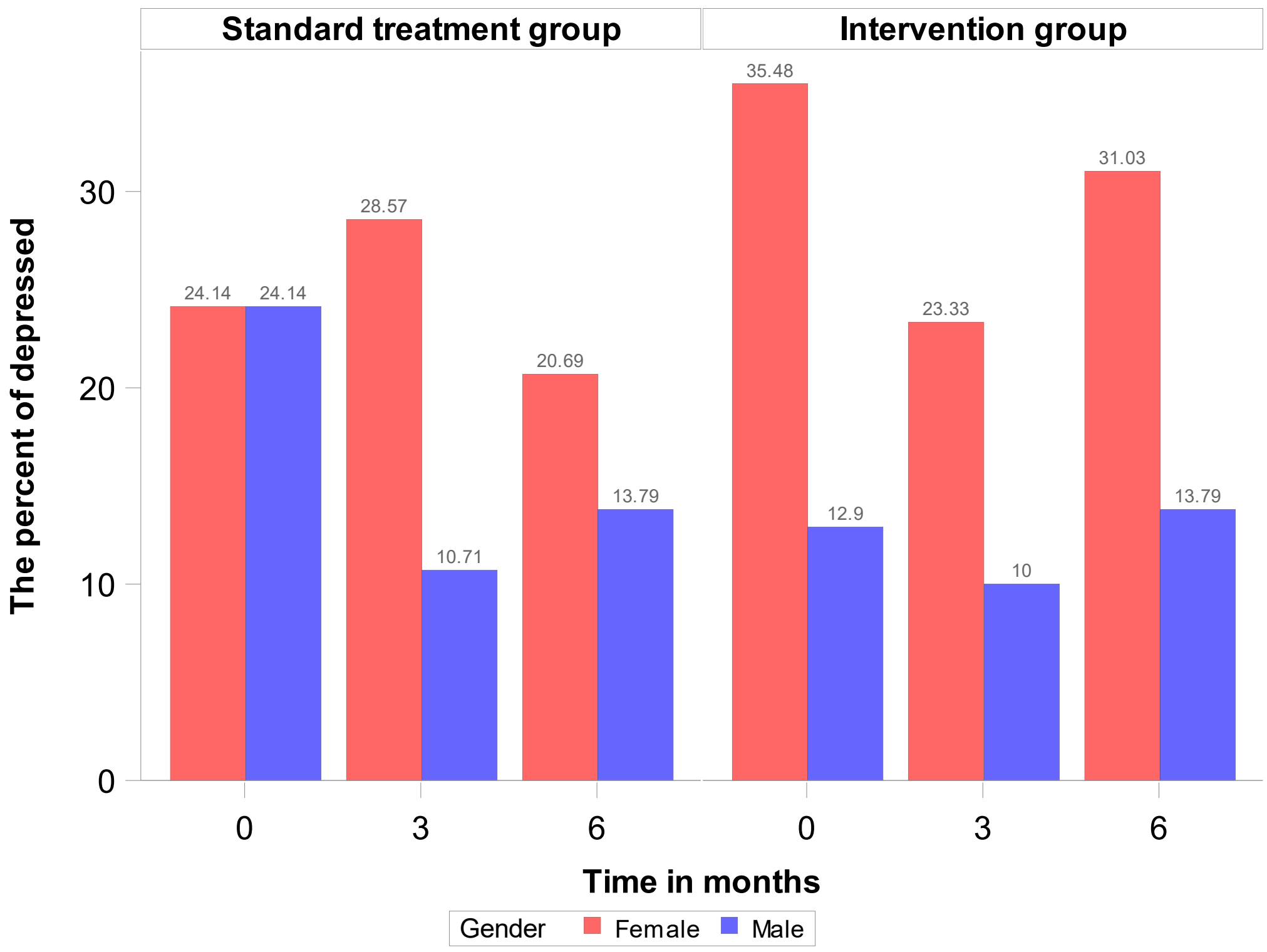

Figure 5. The barplot of the percent of patients with depression in each randomization arm, at 0, 3 and 6 months of follow-up. The percent of patients with depression per time points is broken down by gender.

Table 15. The count and percent per time point, of the patients with breathing problems in the Standard treatment group. Confidence intervals for proportion of patients with breathing problems are calculated as asymptotic Wald confidence intervals for a binomial value.

Time	Breathlessness (no/yes)			Wald CI
**Frequency**<br>**Percent of the total per time point**	**0**	**1**	**Total**	
**Symptom: breathlessness T1**	12<br>41.38	17<br>58.62	29	(0.4070, 0.7655)
**Symptom: breathlessness T2**	14<br>50.00	14<br>50.00	28	(0.3148, 0.6852)
**Symptom: breathlessness T3**	14<br>48.28	15<br>51.72	29	(0.3354, 0.6991)
**Total**	40	46	86	
**Frequency Missing = 1**				

Table 16. The count and percent per time point, of the patients with breathing problems in the Intervention group. Confidence intervals for proportion of patients with breathing problems are calculated as asymptotic Wald confidence intervals for a binomial value.

Time	Breathlessness (no/yes)			Wald CI
**Frequency**<br>**Percent of the total per time point**	**0**	**1**	**Total**	
	8<br>25.81	23<br>74.19	31	(0.5879, 0.8960)
	11<br>36.67	19<br>63.33	30	(0.4609, 0.8058)
	12<br>41.38	17<br>58.62	29	(0.4070, 0.7655)
**Total**	31	59	90	
**Frequency Missing = 3**				

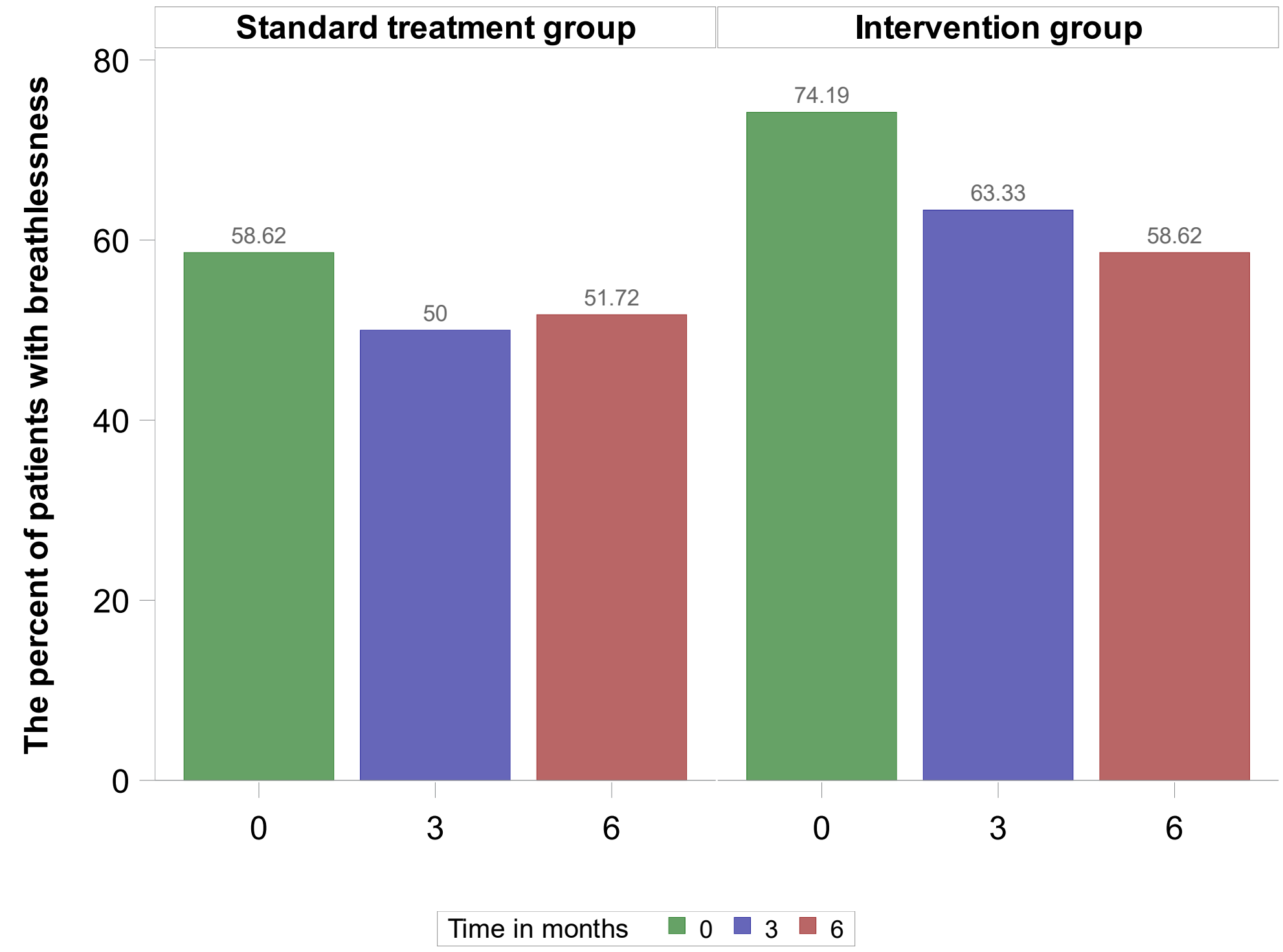

Figure 6. The barplot of the percent of patients with breathing problems in each randomization arm, at 0, 3 and 6 months of follow-up.

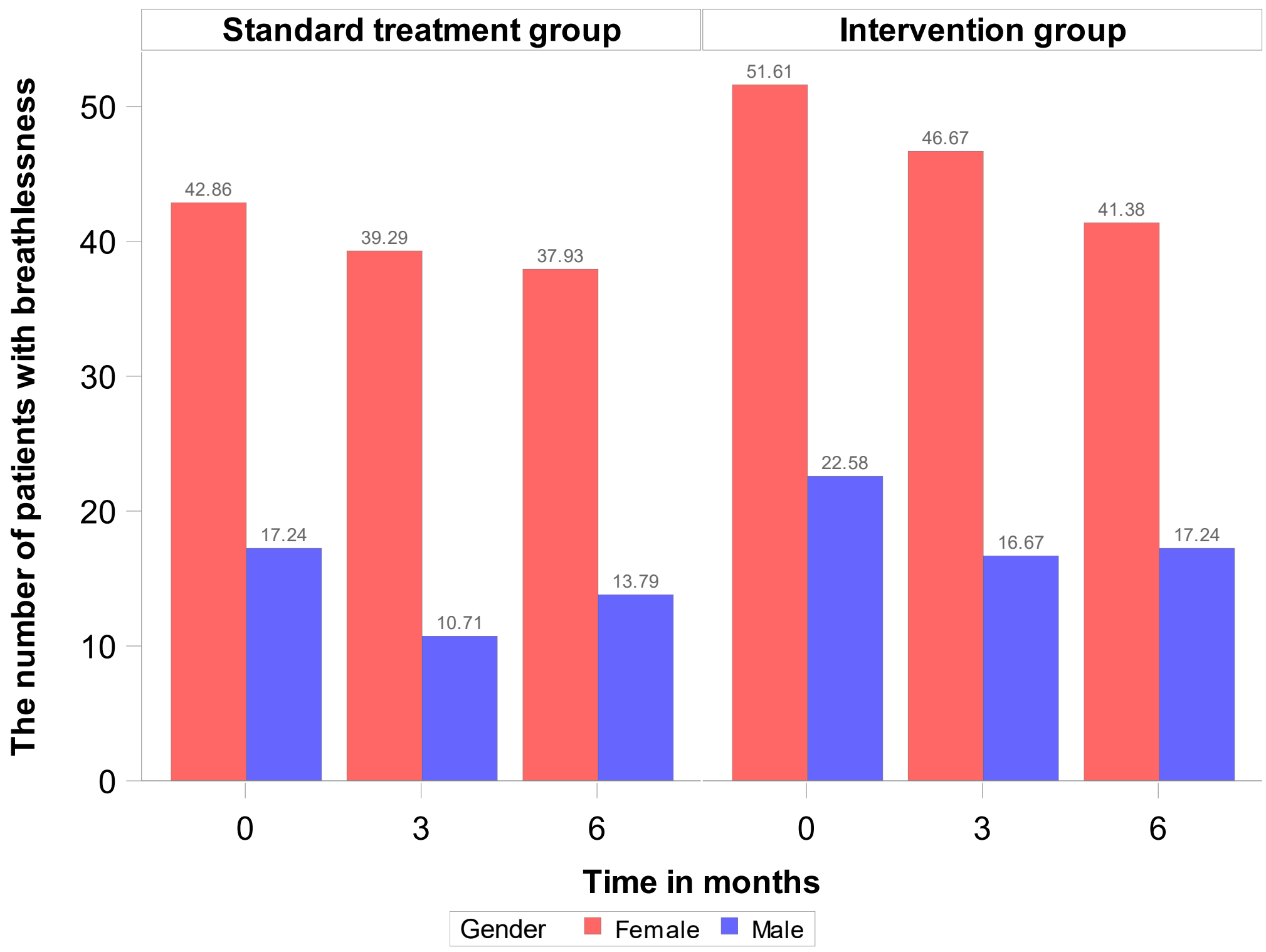

Figure 7. The barplot of the percent of patients with breathing problems in each randomization arm, at 0, 3 and 6 months of follow-up. The percent of patients with depression per time points is broken down by gender.

Table 17. The count and percent at baseline and 6 months of follow-up of the patients that used the rehabilitation app in the Standard treatment group. Confidence intervals for proportion of patients that used the app is calculated as asymptotic Wald confidence intervals for a binomial value.

Time	Rehabilitation app use (no/yes)			Wald CI
**Frequency**<br>**Percent of the total per time point**	**0**	**1**	**Total**	
**App usage T2**	17<br>60.71	11<br>39.29	28	(0.2120, 0.5738)
**App usage T3**	21<br>72.41	8<br>27.59	29	(0.1132, 0.4385)
**Total**	38	19	57	
**Frequency Missing = 1**				

Table 18. The count and percent at baseline and 6 months of follow-up of the patients that used the rehabilitation app in the Intervention group. Confidence intervals for proportion of patients that used the app is calculated as asymptotic Wald confidence intervals for a binomial value.

Time	Rehabilitation app use (no/yes)			Wald CI
**Frequency**<br>**Percent of the total per time point**	**0**	**1**	**Total**	
**App usage T2**	11<br>36.67	19<br>63.33	30	(0.4609, 0.8058)
**App usage T3**	15<br>53.57	13<br>46.43	28	(0.2796, 0.6490)
**Total**	26	32	58	
**Frequency Missing = 4**				
